# Trends in dietary supplement use among athletes selected for doping controls

**DOI:** 10.3389/fnut.2023.1143187

**Published:** 2023-03-15

**Authors:** Fredrik Lauritzen, Astrid Gjelstad

**Affiliations:** ^1^Science and Medicine, Anti-Doping Norway, Oslo, Norway; ^2^Department of Pharmacy, The Faculty of Mathematics and Natural Sciences, University of Oslo, Oslo, Norway

**Keywords:** dietary supplement, doping, sport, athlete, PWO, doping control, sport supplement

## Abstract

**Background:**

Dietary supplements (DS) may be beneficial for athletes in certain situations, whereas incorrect or excessive use may impair performance, pose a risk to the athlete's health and cause positive doping tests by containing prohibited substances. To provide athletes with relevant and tailored information on safe supplement use, a better knowledge about DS trends over time and between sport disciplines are needed.

**Methods:**

This study examines the use of DS among athletes who have participated in doping controls by extracting information derived from 10,418 doping control forms (DCF) collected by Anti-Doping Norway from 2015 to 2019.

**Results:**

Overall, 51% of the DCFs contained information about at least one DS. National level athletes (NLA) more often reported using DS than recreational athletes (RA) (53 vs. 47%, *p* < 0.001). Athletes in strength and power (71%), VO2_max_ endurance (56%) and muscular endurance sports (55%) had the highest proportion of DCFs with information about DS. Medical supplements were the most used supplement category for both genders and across all sports. Dietary supplements with a high risk of containing doping substances were most common among male, RA in strength and power sports. There were small and non-significant year-to-year variations in the prevalence of athletes using DS, while the number of products used concomitantly peaked in 2017 before declining in 2019 (2.30 vs. 2.08, *p* < 0.01). The use of medical supplements and ergogenic substances increased slightly for both NLA and RA from 2015 to 2019, while the use of all other supplement categories declined.

**Conclusion:**

Half of the 10,418 DCFs contained information about DS, with variations within the athlete population. DS with high risk of containing prohibited substances were mostly seen in sport disciplines requiring a high degree of specialization in strength/power, including powerlifting and weightlifting, as well as in some team sports, such as cheerleading and american football.

## Introduction

Adequate nutrition plays a decisive role in optimizing athletic performance (1). A food-first approach is generally recommended, whereas dietary supplements (DS) may occasionally be practical alternatives or additions to meet nutritional goals, treat nutrition deficiencies or in some cases provide a small, but significant ergogenic effect (2, 3). There is no single definition for what constitutes a DS, however, it is often described as “a food, food component, nutrient, or non-food compound that is purposefully ingested in addition to the habitually consumed diet with the aim of achieving a specific health and/or performance benefit” (4).

The global sport nutrition market is a multibillion-dollar industry, expected to nearly double in value by 2030 (5). Correspondingly, athlete's use of DS is reported to be high across sport disciplines (6, 7), and particularly prevalent among elite athletes (8) and athletes in speed, power and endurance-based sports (9). Athlete's motivations for using DS may vary, but are often related to improving athletic performance, improving health and accelerating recovery (6). In addition, athletes frequently report using supplements because “the best athlete use it” (10).

Dietary supplements may be beneficial in certain situations, but if used excessively or incorrectly, they may also have the potential to impair athlete performance (11), damage health and inflict athletes with Anti-Doping Rule Violations (ADRV) by containing substances prohibited for use by athletes under the World Anti-Doping Agency's (WADA) World Anti-Doping Code (12). According to a review of 50 studies which examined the presence of undeclared doping substances in DS frequently used in sports, 28% of the analyzed supplements posed a risk of causing unintentional doping (13). Dietary supplements popular among athletes may also contain declared prohibited substances (14), which could still result in unintentional doping if athletes are not aware of the possible risks associated with such products (15). Recent data suggest that DS use by athletes continue to represent a source of prohibited substances and a major cause of ADRVs (16).

Dietary supplements encompass a large and heterogenous group of products with large variations regarding ingredients, nutritional and performance enhancing effects, health risk and the likelihood that the product contain prohibited substances (7, 17). To develop targeted information for athletes and athlete support personnel on safe supplement use, a better understanding of prevalence and usage patterns of DS across athlete groups and sport disciplines is needed. Substantial research on the use of supplements among athletes has been done [e.g., (6, 7)], however, different data collection methods, athlete populations, and a lack of homogeneity in definitions and categorization of DS complicate direct comparison between studies. There is also a lack of longitudinal studies describing usage patterns over time. No quantitative prevalence data which include multiple sport disciplines since 2003 (18) makes the current landscape of supplement use in the Norwegian athlete population unknown.

Anti-Doping Norway (ADNO) annually collects ~2,800 doping samples divided into 2,000 doping tests of athletes participating in sports organized under the Norwegian Olympic and Paralympic Committee and Confederation of Sports (19). During the doping test, each athlete is required to fill out a doping control form (DCF) including demographic information, athlete level, sport discipline and information about any DS and/or pharmaceutical drug used within the preceding seven days. This study examines the use of DS among athletes who have participated in doping tests by using information derived from DCFs collected by ADNO in the period 2015–2019. Information about pharmaceutical drug use will be presented in a separate study.

## Materials and methods

### Data

Doping control forms from all doping tests where ADNO was the Testing authority (i.e. the Anti-Doping organization that authorized testing on athletes it has authority over) in the period 2015–2019 were included in the study. The material included DCFs retrieved from ADNOs paper archives collected through doping tests of Norwegian athletes performing their sport in Norway or abroad, as well as from athletes from other countries exercising their sport in Norway under the jurisdiction of a Norwegian sport federation organized under the Norwegian Olympic and Paralympic Committee. The number of DCFs retrieved from the paper archives per year was checked against the WADA Anti-Doping Administration and Management System (ADAMS) DCF report to ensure that the DCF paper copies reflected the actual number of doping tests performed in 2015–2019.

The following information from each DCF was manually registered into an electronic database using Microsoft Excel: Year of the doping control, gender, age group (<20; 20–24; 25–29; 30–34; 35–39; ≥40), athlete level {national level athlete [as defined by ADNO (20)]; recreational athlete}, sport discipline, type of test (in-competition (samples collected upon conclusion of competition); out-of-competition) and nationality. Sport disciplines were categorized into the following groups based on physiological characteristics and the risk of doping: Aiming sports, ball and team sports, combat sports, gymnastic sports, muscular endurance sports, other sports, strength and power sports and VO2_max_ endurance sports (19, 21).

Furthermore, it was registered whether the athlete had registered information about the use of any DS during the seven days preceding the doping control (yes; no). The use of DS was defined as registering one or more dietary supplements on the DCF. Dietary supplements were registered by category (medical supplements; sports products; ergogenic substances; mixed products; herbal and other natural products; other products) [modified from Garthe and Maughan (7)], product type (e.g., protein powder; vitamin D; creatine) (Table 1) and product name (not shown).

**Table 1:** Categorization of dietary supplements.

**Category**	**Product type**	**Comments**
Medical supplements	Electrolytes Fish oil/omega 3 Minerals *(e.g., Calcium, iron, magnesium, zinc)* Probiotics Vitamins *(e.g., multi-vitamin, C-vitamin)*	Concentrated sources of micronutrients. Often used to prevent or treat nutritional deficiencies.
Sports products	Protein *(e.g., protein powder, BCAA)* Carbohydrate products *(e.g., sports drink)* Carbohydrate + protein products *(e.g., sports bar, protein shake, mass gainer)*	Provides a practical source of nutrients. Often contain concentrated amounts of macronutrients, or combinations of macronutrients. Come in various forms, including bars, drinks, gels and powder.
Ergogenic substances	Caffeine Creatine Bicarbonate Beta-alanine Nitrate Energy drink *(e.g., Red bull)*	Concentrated sources of single ergogenic substances intended to enhance performance. Comes as tablets, powder or in liquid form. Also include pre-mixed energy drinks containing single, or blends of ergogenic substances.
Mixed products	Pre-workout *(PWO)* Fat burners *(e.g., CLA)* Testosterone boosters *(e.g., ZMA)*	Products containing a blend of ingredients with ergogenic and non-ergogenic properties intended to enhance muscle growth, stimulate metabolism, delay fatigue or in other ways improve exercise performance. The amount of the active biological substance(s) is often unclear, and the declaration of ingredients may be incomplete. Comes as powder or tablets.
Herbal, botanical and similar natural products	Plants, herbs, roots Super greens Mushrooms	Assumed to optimize health. Often marketed as a safer and more “natural” alternative to pharmaceutical drugs.
Other products	Muscle and joint products Homeopathic supplements	Claimed effects varies. Some products aimed at improving health.

All information was registered fully anonymized, thus no identifiers like the athlete's name, address, and exact age, sample code nor date of the control was included in the database.

All statistics in this paper refers to the number of DCFs and not to athletes. Due to the fully anonymized database, it was not possible to merge DCFs from athletes being tested multiple times. Some athletes were tested more than once during the time-period and may have registered the same DS on the DCFs of consecutive doping controls. Consequently, data from athletes with a comprehensive test history may have disproportionally affected the mean values. This limitation is particularly evident in sports disciples consisting of relatively few athletes being tested multiple times. Thus, for comparison of differences in DS use within sport categories and sport disciplines, only groups/disciplines with at least 100 DCFs with information about supplements were included in the analysis.

### Statistics

IBM SPSS Statistics for Windows version 27 was used for data analysis. Descriptive data is presented as mean ± SD for continuous variables and percentages for categorical variables, if not otherwise stated.

Comparison of means between two groups (e.g., gender, athlete level) were examined by using Independent-Samples *t* Tests, while One-Way ANOVA with Tukey *post hoc* test was used for comparisons of three or more groups (e.g., year). The significance level was set to *p* < 0.05.

The study has been approved by the Norwegian Regional Committee for Medical and Health Research Ethics.

## Results

### General population

A total of 10,418 DCFs (males: 76%, *n* = 7,939, females: 24%, *n* = 2,479) from 2015 to 2019 were included in the study. National level athletes (NLA) and recreational athletes (RA) constituted 59% (*n* = 6,184) and 41% (*n* = 4,234) of the DCFs, respectively. Athletes with Norwegian citizenship constituted 94% of the DCFs, while 6% were from athletes with other nationalities. A small majority of the DCFs (54%) were obtained from out-of-competition doping controls.

The material represents athletes from 67 different sport disciplines, of which 25 sport disciplines contributed with ≥100 DCFs in the period.

### Registration of dietary supplements

When including all DCFs from the entire period, half of all DCFs (51%, *n* = 5,287) contained information about at least one DS, of which 21% (*n* = 2,169) contained information about one DS, 13% (*n* = 1,391) about two DS, 8% (*n* = 815) about three DS, 4.5% about four DS and 4.2% of the DCFs contained information about five or more DS.

Females more often than men reported using DS (57%, *n* = 1,417 vs. 49%, *n* = 3,870, *p* < 0.001) and female athletes who entered information about DS on their DCF, used more products concomitantly than males (2.4 ± 1.6 vs. 2.2 ± 1.4, *p* < 0.001).

Doping control forms from NLA more often contained information about DS than DCFs from RA (53 vs. 47%, *p* < 0.001), however, there was no difference when comparing the number of DS used concomitantly between NLA and RA (2.24 ± 1.48 vs. 2.24 ± 1.52, *p* = 0.86). For both groups, the maximum number of DS on a single DCF was 10. Athlete level was a stronger predictor of DS use than gender, as both male and female NLA more often reported using DS than male (50 vs. 47%, *p* < 0.001) and female (61 vs. 50%, *p* < 0.001) RA.

The largest share of supplement users was found among strength and power athletes (71% of DCFs, *n* = 1,251 of 1,758) followed by athletes in VO2_max_ endurance sports (56%, *n* = 1,819 of 3,233) and muscular endurance sports (55%, *n* = 101 of 184) (Table 2), whereas less than half of the DCFs collected in combat sports, other sports, ball and team sports, gymnastic sports and aiming sports contained information about DS.

**Table 2:** Categorization of sport disciplines ranked by the proportion of doping control forms (DCFs) within each sport category which includes information about dietary supplements (DS).

**Sport category**	**DCFs**	**DCFs with DS** ^*^		**Examples of sport disciplines**
	***n***	***%***	***n***	
Strength and power sports	1,758	71.2%	1,251	Athletics sprint and throws, powerlifting, weightlifting etc.
VO2_max_ endurance sports	3,233	56.3%	1,819	Biathlon, canoe, cross-country skiing, cycling, rowing etc.
Muscular endurance sports	184	54.9%	101	Alpine skiing, climbing, sailing etc.
Combat sports	799	47.8%	382	Boxing, fencing, judo, karate, wrestling etc.
Other sports	151	40.4%	61	Air sports, motor sport, underwater sports etc.
Ball and team sports	4,057	39.3%	1,593	Basketball, football, handball etc.
Gymnastic sports	130	37.7%	49	Dancing, Gymnastics, ski jump, snowboard etc.
Aiming sports	106	29.2%	31	Archery, curling, golf, shooting etc.
Total	10,418	50.7%	5,287	

* The proportion of DCFs with information about ≥1 dietary supplement.

For comparison of supplement use across sport disciplines, only disciplines with ≥100 DCFs during the period were included in the analysis. Most frequent use was seen in powerlifting (79% of DCFs, *n* = 512), weightlifting (68%, *n* = 256), speed skating (65%, *n* = 163), athletics (65%, *n* = 454), rowing (63.8%, *n* = 97), cross-country skiing (63.7%, *n* = 630), orienteering (63.0%, *n* = 85) and alpine skiing (62%, *n* = 93) (Table 3). Among the athletes who had registered DS information on the DCF, athletes in powerlifting (3.2 ± 2.0 products per DCF), weightlifting (2.7 ± 1.5), rowing (2.7 ± 1.5), athletics (2.6 ± 1.6), and wrestling (2.6 ± 1.5) had the highest mean use per DCF.

**Table 3:** Prevalence of dietary supplement (DS) use by sport disciplines with ≥100 doping control forms (DCFs).

**Sport discipline**	**Total DCFs**	DCFs with DS^*^	**DS per DCF** ^**^	
	***n***	**%**	**Mean**	**SD**
Powerlifting	649	78.9	3.2	2.0
Weightlifting	375	68.3	2.7	1.5
Speed skating	249	65.5	2.0	1.3
Athletics	695	65.3	2.6	1.6
Rowing	152	63.8	2.7	1.5
Cross-country skiing	989	63.7	2.1	1.1
Orienteering	135	63.0	1.8	1.0
Alpine skiing	150	62.0	2.1	1.1
Kickboxing	190	57.4	2.2	1.4
Wrestling	137	54.0	2.6	1.5
Boxing	166	52.4	2.0	1.5
Judo	102	50.0	2.0	1.2
Biathlon	378	49.7	1.9	1.3
Triathlon	173	49.1	1.8	1.1
Cycling	735	48.7	2.5	1.8
Icehockey	657	46.4	1.9	1.1
American football	251	45.0	2.1	1.3
Cheerleading	154	42.9	2.2	1.3
Swimming	244	42.2	1.8	1.2
Basketball	380	41.8	1.9	1.3
Floorball	190	39.5	1.6	1.1
Handball	614	37.5	1.9	1.2
Football	1,358	36.6	1.9	1.4
Volleyball	298	31.2	1.7	1.1
Taekwondo	105	22.9	1.3	0.5
Total^#^	9,526	51.5	2.1	1.2

The order of the sport disciplines is sorted by share of DCFs with information about at ≥ DS.

* The proportion of DCFs with information about ≥1 DS.

** The mean number of supplements reported on the DCF of athletes using ≥1 DS.

# Total mean values of DCFs of all sport disciplines with ≥100 DCFs.

### Type of dietary supplement

Medical supplements were the most used supplement category for both female (49% of all DCFs contained information about this supplement category) and male athletes (34%), followed by sports products (female: 11%; male: 15%) and ergogenic substances (female: 12%; male: 13%). Among athletes who reported supplement use, 86% of female athletes and 70% of male athletes used at least one type of medical supplement. Male athletes using DS, more often registered sports products (31%) and ergogenic substances (28%) compared to females (19.8 and 20.3%, respectively). Only 4% of the DCFs from female DS users contained information about mixed products, whereas 12% of the DCFs of male DS users contained information about one or more products in this category.

Among NLA, 42% of all DCFs contained information about medical supplements compared to 30% in RA. Recreational athletes more frequently used mixed products (8.4 vs. 2.6%) and sports products (18 vs. 12%) than NLA, while there were no clear differences between RA and NLA in the use of ergogenic substances (13.4 vs. 12.7%), other products (0.6 vs. 0.4%) or natural products (2.8 vs. 3.6%).

**Figure 1:**
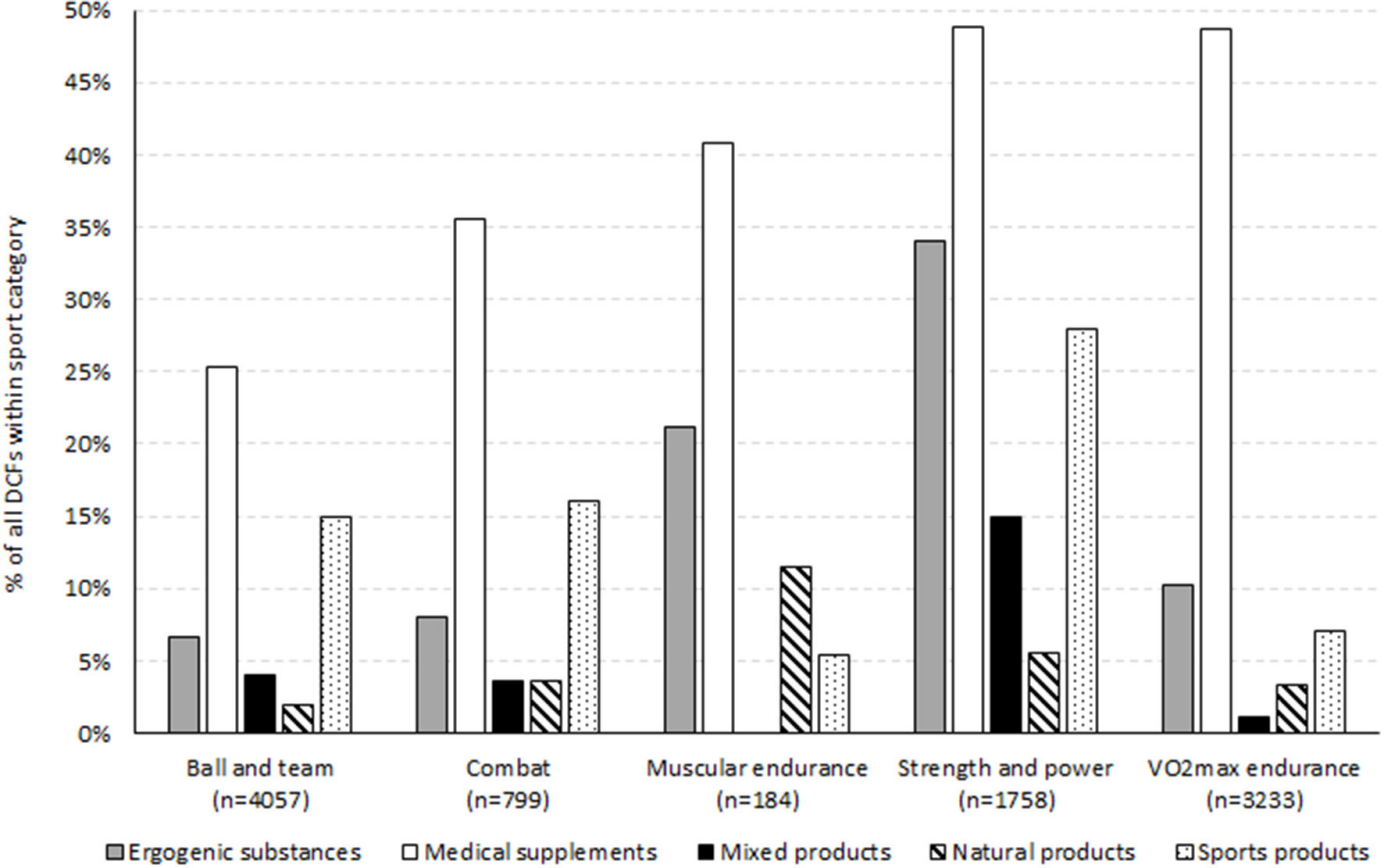
Use of dietary supplements (DS) by sport category. The DS category Other products was registered on <1% of the DCFs in each sport category and are not shown. The sport categories aiming sports, gymnastic sports and other sports contained <100 DCFs and are not included in the figure. n represents the total number of DCF from athletes in the respective sport category.

Aiming sports (*n* = 31), gymnastic sports (*n* = 49) and other sports (*n* = 61) all had <100 DCFs which included DS information and were thus excluded when comparing supplement use across sport categories. Medical supplements were the most prevalent dietary supplement category in all sport categories. Strength and power athletes had the most frequent use of all supplement categories, except for natural products, which were most often used in muscular endurance sports (Figure 1). Strength and power athletes and muscular endurance athletes more often used ergogenic substances (34 and 21% of total DCFs, respectively) than any of the other groups (≤10%). Mixed products were only used to a significant degree in strength and power sports (15 vs. 0–4% in the other sport categories), but with large variations between disciplines within a sport category.

The highest use of medical supplements was seen in cross-country skiing (61% of DCFs), orienteering (57%), athletics (55%), rowing (48.0%) and biathlon (47.9%) (Table 4). Sport products were particularly common in powerlifting (48%), while ergogenic substances were used across sport disciplines requiring various physiological properties but where many are recognized by elements of anaerobic, high power output, such as powerlifting (48%), weightlifting (34%), speed skating (28%), rowing (27%) and alpine skiing (26%). Mixed products were frequently used in powerlifting (27%), cheerleading (16%), American football (15%), weightlifting (13%), kickboxing (5.8%), ice hockey (5.5%) and basketball (5.5%). However, in all other disciplines with ≥100 DCFs, the prevalence of mixed products was below five percent.

**Table 4:** Use of dietary supplements (DS) category by sport disciplines with ≥100 doping control forms (DCFs).

**Sport discipline**	Medical supplements^*^(%)	Sports products^*^(%)	Ergogenic substances^*^(%)	Mixed products^*^(%)	Natural products^*^(%)	Other products^*^(%)
Alpine skiing	46.0	4.7	26.0	0.0	14.0	0.0
American football	21.5	21.5	15.1	14.7	0.4	1.6
Athletics	55.4	6.8	21.9	3.3	6.9	1.0
Basketball	24.2	18.2	8.7	5.5	2.4	0.8
Biathlon	47.9	1.1	0.5	0.0	4.5	0.0
Boxing	39.2	21.7	6.0	4.8	2.4	0.6
Cheerleading	19.5	16.2	13.6	15.6	2.6	0.6
Cross-country skiing	60.8	2.8	8.0	0.0	2.7	0.1
Cycling	42.3	8.4	9.5	2.9	2.6	0.3
Floorball	23.7	13.7	8.4	4.2	1.1	1.2
Football	27.4	10.0	5.4	1.8	1.9	0.1
Handball	24.9	15.8	4.4	2.0	2.8	0.5
Ice hockey	25.7	20.9	8.4	5.5	1.7	1.2
Judo	27.5	20.6	18.6	3.9	3.9	1.0
Kickboxing	45.3	20.0	6.8	5.8	6.3	0.5
Orienteering	57.0	5.2	5.2	0.0	2.2	0.0
Powerlifting	43.1	48.2	47.5	26.7	5.2	0.9
Rowing	48.0	23.7	27.0	0.0	6.6	0.0
Speed skating	45.4	5.6	27.7	1.2	0.4	0.0
Swimming	29.9	10.7	9.4	0.8	2.5	0.0
Taekwondo	15.2	4.8	2.9	1.0	0.0	0.0
Triathlon	34.1	8.1	9.8	1.7	6.4	0.0
Volleyball	21.5	7.0	5.0	2.0	1.3	1.0
Weightlifting	46.4	28.5	34.4	13.3	4.0	0.5
Wrestling	43.1	13.1	10.2	3.6	5.1	2.2
**Mean**	**36.5**	**14.4**	**13.3**	**4.7**	**3.6**	**0.6**

Sport disciplines are ranked in alphabetical order. Percentages are presented as proportion of DCFs per sport discipline with information about each DS group. A color scale is used to visualize the share of DCFs in a sport discipline with information about a given DS group.

* The proportion of DCFs with information about ≥1 dietary supplement in the respective supplement category.

In total 43 different product types were registered on the DCFs, of which 20 different products were found on a minimum of 100 DCFs (Table 5). The majority of the DCFs included information about maximum one product per product category, although there were some few examples where an athlete used multiple different products from the same product category, for example multiple PWO. The top three products were all medical supplements (fish oil/omega-3, multivitamins and magnesium). Fish oil/omega-3 and multivitamins were registered on 18 and 14% of all DCFs, respectively. Creatine was the most used ergogenic substance and protein powder the most registered sports product. Pre-workout supplements (PWO) was found on 3% of the DCFs overall. Large variations were found on the prevalence of most product types across sport categories (Table 5).

**Table 5:** The most used dietary supplements (DS) per sport category.

**Product type**	**Supplement category**	**All DCFs (*****n*** = **10,418)**		**Ball and team**		**Combat**		**Muscular endurance**		**Strength and power**		**VO2**_**max**_ **endurance**	
		***n***	%^*^	***n***	%^*^	***n***	%^*^	***n***	%^*^	***n***	%^*^	***n***	%^*^
Fish oil/omega-3	Medical	1,900	18.2	453	11.2	124	15.5	43	23.4	389	22.1	839	26.0
Multivitamins	Medical	1,472	14.1	354	8.7	124	15.5	7	3.8	407	23.2	543	16.8
Magnesium	Medical	853	8.2	228	5.6	65	8.1	9	4.9	256	14.6	276	8.5
Creatine	Ergogenic	825	7.9	188	4.6	41	5.1	34	18.5	484	27.5	53	1.6
Protein powder	Sport	782	7.5	312	7.7	75	9.4	4	2.2	301	17.1	66	2.0
Vitamin D	Medical	743	7.1	163	4.0	48	6.0	34	18.5	144	8.2	335	1.4
Vitamin C	Medical	713	6.8	226	5.6	49	6.1	15	8.2	144	8.2	267	8.3
Iron	Medical	523	5.0	78	1.9	26	3.3	2	1.1	123	7.0	288	8.9
BCAA/AA	Sport	458	4.4	135	3.3	45	5.6	3	1.6	222	12.6	47	1.5
Carbs + protein	Sport	363	3.5	168	4.1	28	3.5	4	2.2	84	4.8	64	2.0
Caffeine	Ergogenic	321	3.1	61	1.5	6	0.8	2	1.1	86	4.9	163	5.0
PWO	Mixed	308	3.0	121	3.0	18	2.3	0	0.0	149	8.5	8	0.2
Other vitamines	Medical	281	2.7	75	1.8	14	1.8	7	3.8	83	4.7	98	3.0
Beta alanine	Ergogenic	253	2.4	4	0.1	13	1.6	15	8.2	71	4.0	148	4.6
Calcium	Medical	252	2.4	24	0.6	20	2.5	0	0.0	78	4.4	130	4.0
Plants	Natural	233	2.2	51	1.3	27	3.4	0	0.0	77	4.4	71	2.2
Carbohydrate p.	Sport	214	2.1	82	2.0	10	1.3	0	0.0	36	2.0	78	2.4
Testobooster	Mixed	187	1.8	33	0.8	11	1.4	0	0.0	128	7.3	9	0.3
Zinc	Medical	165	1.6	24	0.6	14	1.8	0	0.0	48	2.7	78	2.4
Probiotics	Medical	158	1.5	16	0.4	4	0.5	4	2.2	32	1.8	100	3.1

The sport categories Aiming sports, Gymnastic sports and Other sports each contained < 100 doping control forms (DCFs) with information about DS and are not shown.

* The proportion of DCFs with information about a specific product. ^**^The proportion of DCFs with information about a specific product when only including DCFs with ≥1 DS.

AA, Amino acids; BCAA, Branced Chain Amno Acids; Carbs, carbohydrates; P, products; PWO, Pre-workout.

### Use of dietary supplements over time

When examining all DCFs together, the use of dietary supplements varied across the period, however changes over time in the share of DCFs per year across with information about at least one DS were small and non-significant (*p* < 0.095) (Table 6). The number of mean products used concomitantly by supplement users peaked in 2017 with 2.36 DS per DCF, before declining to 2.30 DS in 2018 and 2.08 DS in 2019 (*p* < 0.01). The trend persisted also when split into RA and NLA.

**Table 6:** Use of dietary supplements (DS) over time among recreational athletes and National level (NL) athletes.

	**All DCFs**		**Recreational athletes**		**NL athletes**	
	**% DCFs** ^*^	**DS per DCF** ^**^	**% DCFs** ^*^	**DS per DCF** ^**^	**% DCFs** ^*^	**DS per DCF** ^**^
2015	50.0	2.25	46.7	2.25	51.6	2.25
2016	49.1	2.23	46.1	2.20	50.7	2.25
2017	52.0	2.36	49.2	2.34	53.5	2.37
2018	52.8	2.30	50.8	2.34	54.5	2.26
2019	49.9	2.08	44.9	2.09	55.8	2.08
**Total**	**50.7%**	**2.24**	**47.4 %**	**2.24**	**53.0%**	**2.24**

* The proportion of doping control forms (DCFs) with information about 1 DS.

** The mean number of supplements reported on the DCF of athletes using ≥1 DS.

The prevalence of specific supplement categories among RA and NLA to some degree varied over time (Table 7). Medical supplements remained the most used supplement category throughout the period for both RA and NLA. There were small increases in the use of medical supplements and ergogenic substances over time while less frequent use was seen for sports products, mixed products, natural products and other products.

**Table 7:** Proportion of doping control forms (DCF) with registered information about each dietary supplement category over time.

**Product category**	**Athlete level**	**All DCFs**		**2015**	**2016**	**2017**	**2018**	**2019**
		***n***	%^*^	%^*^	%^*^	%^*^	%^*^	%^*^
Medical supplements	Recreational	1,288	30.4	27.4	29.9	29.7	35.0	29.1
	National	2,620	42.4	38.9	40.8	42.7	46.0	44.6
Sports products	Recreational	757	17.9	22.0	17.7	22.4	16.9	13.9
	National	726	11.7	13.6	12.0	12.0	10.7	9.8
Ergogenic substances	Recreational	568	13.4	11.2	12.5	12.3	15.3	14.2
	National	786	12.7	12.5	12.6	12.4	12.2	14.1
Mixed products	Recreational	356	8.4	8.8	8.5	9.0	9.2	7.2
	National	158	2.6	3.2	2.0	2.7	2.9	2.1
Natural products	Recreational	118	2.8	3.7	2.1	3.4	3.5	1.8
	National	224	3.6	4.1	3.6	3.8	3.6	2.8
Other products	Recreational	27	0.6	0.9	0.4	0.4	0.8	0.6
	National	24	0.4	0.6	0.4	0.5	0.3	0.2

* The proportion of DCFs with information about ≥1 dietary supplement in the respective year.

## Discussion

The present study examines DS use among athletes selected for doping control by ADNO in the period 2015–2019. The study describes DS prevalence and trends among athletes representing 67 sport disciplines by extracting information declared by athletes from 10,418 DCFs.

There is consensus among nutritional experts that a food-first approach is recommended and that a well-balanced and varied diet in most cases is adequate for athletes to meet their nutritional demands (3, 22). Nevertheless, the use of DS among athletes has consistently been reported to be high. Two reviews examining the periods 2003–2017 (7) and 2017–2022 (6) found that 40–70% and 10–100% of athletes use DS, respectively, depending on the type of sport, definition of dietary supplements and level of competition. A study surveying Norwegian elite athletes in 2003 found that 51% of male athletes and 54% of female athletes reported using one or more supplement (18). A study based on information from DCFs collected from Danish registered testing pool (i.e., elite) athletes in 2014, found that 85.0% of male and 92.6% of female athletes declared using one or more DS (23).

Overall, half of the DCFs examined in the present study contained information about one or more DS. There were, however, variations within the athlete population, and to some degree from year to year. Among females there were both a larger proportion of athletes using DS than among male athletes, and females had a higher mean consumption of DS. A higher prevalence among female athletes has also been found in another Nordic country (23), while other studies don't support this finding (8, 24, 25). Athlete level was though a stronger predictor of DS use than gender, as both male (52.5%) and female NLA (60.1%) more often used supplements than male and female RA, which is in line with other studies reporting that elite athletes are more frequent users of DS than non-elite athletes (8).

It has been suggested that the culture generated by a particular sport influences supplement use among its athletes (10). Indeed, sport discipline significantly influenced the prevalence and type of products used. Athletes in so-called CGS sports (i.e., sports where the results are measured in centimeters, grams and seconds), more often used supplements and had a higher mean consumption per athlete, compared to sports requiring more complex physical properties, with larger emphasis on technical and tactical skills. The largest share of supplement users and the highest mean consumption per athlete was seen in individual sports characterized by performances based on one or few bio-motor abilities and which require a high degree of specialization in either maximum strength/power or aerobic or anaerobic capacity, such as example powerlifting, weightlifting, speed skating, athletics and rowing. A lower use of DS in team sports than individual sport is consistent with other studies (9, 24). According to the vulnerability thesis, highly specialized sports are more vulnerable of using prohibited performance enhancing substances or methods (26). The results presented here suggests that athletes in sports with a high degree of specialization are also more likely to seek legal performance enhancing by using DS.

The high prevalence of DS use by athletes has raised concern among Anti-Doping organizations (27), as such products may contain prohibited substances [e.g., (28)], and thus may lead to ADRVs (16, 29). Furthermore, it has been proposed that athletes using DS have more positive attitudes toward doping (30), that the use of supplements positively correlates with doping intentions and behaviors (31), and that athletes engaged in legal performance enhancement through supplement use may be at risk for transition toward doping (32, 33). However, considering the widely heterogenic product group that is DS, it may not be appropriate to include all product types when discussing DS in relation to doping. As shown in the present study, athletes use a wide variety of supplements, and although prohibited substances has been found in all types of supplements (34, 35), many of the most commonly used products among athletes could be considered low risk in terms of the likelihood that they contain prohibited substances. In particular, multi-ingredient pre-workout products, and products making claims of performance enhancement, muscle building or weight loss are known to be associated with high risk of contamination or containing declared prohibited substances (14, 16, 17, 36). In this study, high risk supplements are mostly confined within the mixed products category. On average 4.7% of all DCFs contained information about one or more mixed product, with a prevalence range between 0 and 27% among the 25 sport disciplines which contributed with 100 DCFs or more to the total data material. The most frequent use of mixed products was found in powerlifting, cheerleading, american football and weightlifting. Interestingly, these sports are frequently viewed as high risk disciplines and rank among the top sports with the most global (37) and national ADRVs (19).

In a previous study, we showed that of all ADRVs associated with DS in ADNO's national testing program, 63% were from athletes in ball and team sports while 15% were from strength and power sports and the remaining 22% from VO_2_max endurance sports, other sports, combat sports or gymnastic sports (16). When we see those findings in the context of the data presented here, it does not appear that the use of DS in itself is associated with a high risk of ADRVs caused by DS. As shown in this study, ball and team sports had the second lowest proportion of athletes using supplements when comparing DS prevalence among the sport categories. Rather, the prevalence of high-risk products is likely of greater importance. Indeed, in addition to powerlifting, weightlifting and kickboxing, the team sports cheerleading, American football, ice hockey, basketball and boxing were the only disciplines with a higher use of mixed products than the total average of the 25 disciplines with 100 or more DCFs.

Across the time period, the annual total share of supplement users ranged from 45 to 51% among RA, and 51–56% among NLA, with a small but not uniform increase over time among NLA but not RA. A study on DS use among Norwegian elite athletes from 2003 found that 53% of athletes used one or more DS (18). This is comparable to usage rates in the period 2015–2019. Care should be made when comparing studies due to different populations and methodology, however, the apparent stable prevalence in DS use over time in the present study is somewhat surprising given the explosive annual growth to the sport nutrition market and the warned negative impacts this may have on athletes (27). Unfortunately, the lack of longitudinal studies makes it difficult to compare and assess whether the apparent stable prevalence presented here is representative for other countries.

The potential benefits or negative impacts of supplement use have not been the focus of this paper; however, athletes should be made aware that DS have the potential to cause adverse health effects and impair performance. In general, intake of dietary supplements by healthy individuals in the recommended daily dose is safe, but not without risk (38). An estimated 23 000 emergency visits in the United States are attributed to adverse events related to DS (39). A particularly high health risk seems to be related to products making claims about weight loss and increased energy. Furthermore, there has been several reports of illness and even death attributed to synthetic stimulants (40), which have been persistently found in some weight loss and PWO supplements (16, 41–43). These findings emphasize the importance that athletes who wish to use DS should assess the need, assess the risk and assess the consequences of using any specific DS (12, 44).

## Conclusion

Overall, about half of 10,418 DCFs collected by ADNO in 2015–2019 contained information about at least one DS, with variations within the athlete population. Medical supplements, such as vitamins and minerals, and sports products, such as protein powder and BCAA, were the most used DS. Use of products with a high risk of containing prohibited substances were mostly seen in sport disciplines requiring a high degree of specialization in strength/power, including powerlifting and weightlifting, as well as in some team sports, including cheerleading and American football. The prevalence of DS use was relatively stable across the time period.

Data on supplement use based on athletes' self-declaration in conjunction with doping controls should be interpreted with caution, as athletes may perceive these situations as stressful and may forget to declare supplements they have been taking (7). Also, declaration of substances is limited to 7-days prior to testing and only includes athletes selected for doping control. On the positive side you get a 100% response rate as registration of DS use on the DCF is mandatory. Thus, DCF records may provide a unique data source on supplement use among athletes in a national testing pool.

More longitudinal studies are needed to examine trends within the same athlete population. Also, research should focus on specific product types and not DS in general. For Anti-Doping organizations, more information about prevalence and motivations for using mixed products would be of interest.

## Data availability statement

The raw data supporting the conclusions of this article will be made available by the authors, without undue reservation.

## Ethics statement

The studies involving human participants were reviewed and approved by Norwegian Regional Committee for Medical and Health Research Ethics. Written informed consent from the participants' legal guardian/next of kin was not required to participate in this study in accordance with the national legislation and the institutional requirements.

## Author contributions

FL and AG contributed to conception and design of the study, performed the statistical analysis, contributed to manuscript revision, and approved the submitted version. AG organized the database. FL wrote the first draft of the manuscript. Both authors contributed to the article and approved the submitted version.
